# The Right Time to Happen: Play Developmental Divergence in the Two *Pan* Species

**DOI:** 10.1371/journal.pone.0052767

**Published:** 2012-12-26

**Authors:** Elisabetta Palagi, Giada Cordoni

**Affiliations:** 1 Museo di Storia Naturale e del Territorio, Università di Pisa, Pisa, Italy; 2 Istituto di Scienze e Tecnologie della Cognizione, Consiglio Nazionale delle Ricerche, Roma, Italy; VIB & Katholieke Universiteit Leuven, Belgium

## Abstract

Bonobos, compared to chimpanzees, are highly motivated to play as adults. Therefore, it is interesting to compare the two species at earlier developmental stages to determine how and when these differences arise. We measured and compared some play parameters between the two species including frequency, number of partners (solitary, dyadic, and polyadic play), session length, and escalation into overt aggression. Since solitary play has a role in developing cognitive and physical skills, it is not surprising that chimpanzees and bonobos share similar developmental trajectories in the motivation to engage in this activity. The striking divergence in play developmental pathways emerged for social play. Infants of the two species showed comparable social play levels, which began to diverge during the juvenile period, a ‘timing hotspot’ for play development. Compared to chimpanzees, social play sessions in juvenile bonobos escalated less frequently into overt aggression, lasted longer, and frequently involved more than two partners concurrently (polyadic play). In this view, play fighting in juvenile bonobos seems to maintain a cooperative mood, whereas in juvenile chimpanzees it acquires more competitive elements. The retention of juvenile traits into adulthood typical of bonobos can be due to a developmental delay in social inhibition. Our findings show that the divergence of play ontogenetic pathways between the two *Pan* species and the relative emergence of play neotenic traits in bonobos can be detected before individuals reach sexual maturity. The high play motivation showed by adult bonobos compared to chimpanzees is probably the result of a long developmental process, rooted in the delicate transitional phase, which leads subjects from infancy to juvenility.

## Introduction

Chimpanzees (*Pan troglodytes*) and bonobos (*Pan paniscus*), the humans' closest living primate relatives, differ in multiple aspects of social behavior including aggression [1], conflict management [2], [3], sex [4], cooperation [5], and play [6]. Such differences have been attributed to different neurobiological systems [7], ecological pressures [8], and heterochronic mechanisms [9], which are changes in time of development respective to the ancestral ontogenies [10]. Parker and McKinney [10] pointed out that heterochronic mechanisms can produce both an overdevelopment and an underdevelopment. The underdevelopment, historically defined as pedomorphosis, can be reached via three different timing processes: progenesis (an early termination of development), postdisplacement (a late starting of development), and neoteny (a slowing down in the developmental rate) [11], [10]. Neoteny works throughout all phases of ontogeny and can produce an underdeveloped organism if onset and offset time are unchanged respect to the ancestral organism. The difference of some behavioral and morphological traits between the two *Pan* species are ascribed by many authors to neotenic processes [6], [12]–[16]. One of the main behaviors revealing the neotenic nature of a species is play, an activity strictly linked to the immature phase in most mammals [17]–[20]. Palagi [6] demonstrated that bonobos, compared to chimpanzees, maintain higher levels of playful motivation as adults. Given the evidence for developmental slowing down in bonobo play, it is interesting to compare the two *Pan* species at earlier developmental stages to determine how and when these differences in adult play arise in ontogeny. We hypothesize that the ontogenetic divergence of the two *Pan* species in play behavior occurs before animals reach sexual maturity and, more specifically, from infancy to juvenility. Moreover, if the age-related play divergence between immature bonobos and chimpanzees is due to the higher social tolerance levels showed by the former [15], we expect that this divergence involves more social than solitary play. Furthermore, within social play the divergence between infants and juveniles of the two species is mainly expected in the tolerance propensity during play fighting (or Rough&Tumble, which is characterized by strong physical contact) and polyadic play (when individuals have to manage a higher number of partners all interacting within a single session).

## Results

### Solitary play

A solitary play session started when an individual performed the first play behavioral pattern (see Table 1). If the bout started again after a delay of 10-s it was counted as a new play session. We distinguished object (the animal shakes, dangles, throws, an object of its environment in solitary way) and acrobatic sessions (the animal performs patterns such as pirouetting, somersaulting, headshaking, jumping, and running) for solitary play.

**Table 1:** Play behavioral patterns recorded during the observation sessions of both chimpanzee and bonobo groups.

Locomotor-Rotational play	Definition
Acrobatic Play	An animal climbs, jumps, and dangles from supports in its environment (e.g., branches, ropes) in solitary or social way (animals climb, jump, and dangle together and concurrently often on the same support).
Pirouetting	An animal performs rolling over either on the ground or on vertical supports in solitary or social way (animals roll in contact hanging on the same vertical support)
Play recovering a thing	An animal chases playmate and attempts to grab object carried by it
Play running	An animal runs alone (solitary play) or chases play partner (social play)
Somersault	An animal flips over either on the ground or on vertical supports in solitary or social way (animals flip in contact)
Play jumping	An animal can solitarily jump on the substrate (ground, platforms, rocks, trunks)
Play sliding down	An animal slides down. The sliding down pattern can be done on a slippery surface or on an inclined plane.
Headshaking	An animal shakes its head laterally. Head shaking can be performed also when the animal is upside down
**Play fighting**	
Play biting	An animal gently bites the playmate
Play brusque rushing	An animal jumps with its four limbs on playmate
Play pushing	An animal pushes playmate either with its hands or feet
Play pulling	An animal pulls a playmate with its hand
Play retrieving	An animal holds playmate to prevent its flight
Play slapping	An animal slaps any part of playmate's body
Play stamping	An animal jumps on a playmate with its feet
**Other play patterns**	
Full play face	Playful facial display: mouth is opened with upper and lower teeth exposed. It can be performed both during solitary and social play sessions
Object play manipulation	An animal shakes, dangles, throws, an object of its environment in solitary or social way (when the action is directed to a playmate; the pattern does not imply any kind of contact between the two animals)
Play face	Playful facial display: mouth is opened with only lower teeth exposed. It can be performed both during solitary and social play sessions
Tickle	An animal contacts the partner's body with its mouth or hands

**Figure 1:**
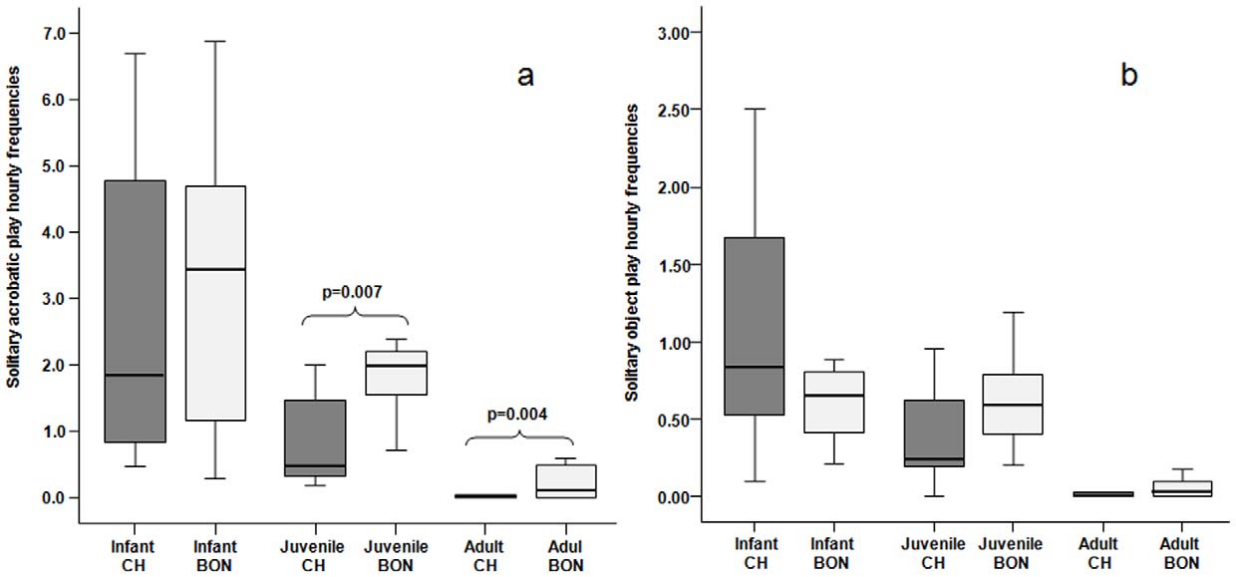
Distribution of solitary play. Hourly frequency of solitary acrobatic play (a) and solitary object play (b) performed by infants, juveniles, and adults of the two *Pan* species. Solid horizontal lines indicate medians; length of the boxes corresponds to inter-quartile range; thin horizontal lines indicate range of observed values. Only the significant differences are reported.

For both species, we found a negative correlation between the acrobatic play levels and the subjects' ages (chimpanzees: Spearman r_s_ = −0.767, N = 36, p = 0.0001, bonobo r_s_ = −0.778, N = 34, p = 0.0001). Infant bonobos and chimpanzees did not differ in their acrobatic play rates (Mann-Whitney U = 19, n_chimp_ = 8, n_bon_ = 7, n.s.); while juvenile and adult bonobos showed higher levels of acrobatic play than chimpanzees (juveniles: U = 3.00, n_chimp_ = 7, n_bon_ = 6, p = 0.007; adults: U = 109.00, n_chimp_ = 21, n_bon_ = 21, p = 0.004) (Figure 1a).

For both species, we found a negative correlation between the object play frequency and the subjects' ages (chimpanzees: r_s_ = −0.720, n = 36, p = 0.0001; bonobo r_s_ = −0.711, n = 34, p = 0.0001). Infants, juveniles, and adults of the two species did not differ in their object play rates (infants, U = 27, n_chimp_ = 8, n_bon_ = 7, n.s.; juveniles, U = 18.00, n_chimp_ = 7, n_bon_ = 6, n.s.; adults: U = 158.50, n_chimp_ = 21, n_bon_ = 21, n.s.) (Figure 1b).

### Social play

A social play session started when an individual directed any playful pattern towards a fellow and ended when the playmates stopped their activities or one of them moved away [21]. For each play session we recorded: i) the identity and the number of playmates, ii) the play patterns performed and their chronological sequence iii) the context in which play took place (e.g. feeding, sexual). Within social play, we distinguished between Locomotor-Rotational play (including play recovering a thing, play run, pirouetting, sliding down, see Table 1), when a session was characterized by the absence of any kind of physical contact between the playmates [22], [23], and play fighting (including biting, pushing, pulling, slapping, stamping, retrieving, brusque rushing; see Table 1), when the participants exhibited physical contact.

#### Play frequency

The Locomotor-Rotational play levels negatively correlated with the playmates' ages for either chimpanzees (r_s_ = −0.779, n = 36, p = 0.000001) or bonobos (r_s_ = −0.832, n = 34, p = 0.000001). The play fighting rates negatively correlated with the chimpanzees' ages (r_s_ = −0.842, n = 36, p = 0.000001) but not with the bonobos' ages (r_s_ = -0.394, n = 34, n.s.).

**Figure 2:**
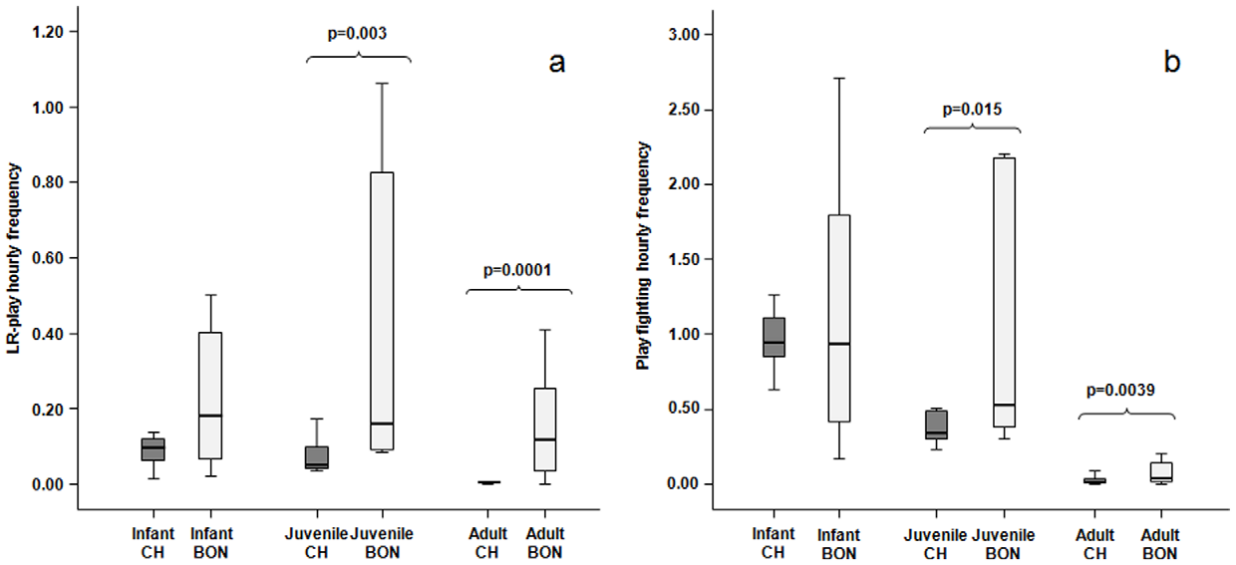
Distribution of social play. Hourly frequency of locomotor-rotational play (a) and play fighting (b) performed by infants, juveniles, and adults of the two *Pan* species. Solid horizontal lines indicate medians; length of the boxes corresponds to inter-quartile range; thin horizontal lines indicate range of observed values. Only the significant differences are reported.

Infant Locomotor-Rotational play and play fighting levels did not differ between the two species (LR-play: U = 16.00; n_chimp_ = 8; n_bon_ = 7; n.s.; play fighting: U = 24.00; n_chimp_ = 8; n_bon_ = 7; n.s.). Conversely, juvenile and adult Locomotor-Rotational play and play fighting rates were higher in bonobos than in chimpanzees (juveniles: LR-play, U = 1.50, n_chimp_ = 7, n_bon_ = 6, p = 0.003; play fighting: U = 4.50, n_chimp_ = 7, n_bon_ = 6, p = 0.015; adults: LR-play, U = 15.0, n_chimp_ = 21, n_bon_ = 21, p = 0.00000021; play fighting: U = 106.0, n_chimp_ = 21, n_bon_ = 21, p = 0.0039) (Figure 2a and 2b).

**Figure 3:**
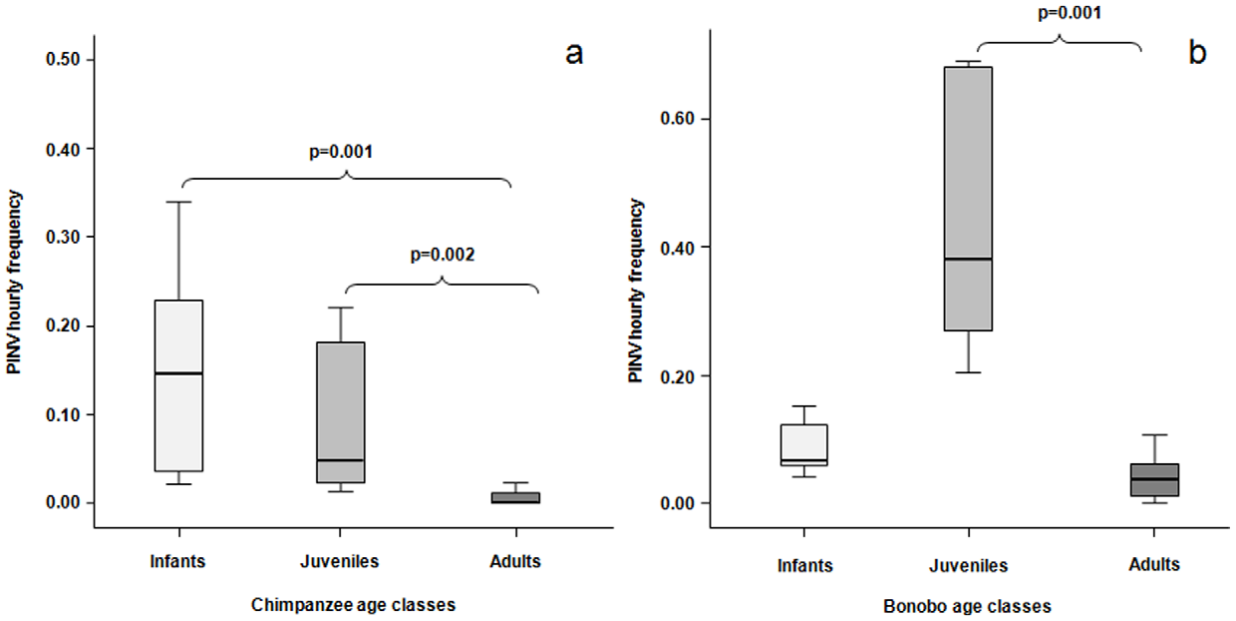
Play invitations in bonobos and chimpanzees. Hourly frequency of play invitations performed by the three age classes of chimpanzees (a) and bonobos (b). Solid horizontal lines indicate medians; length of the boxes corresponds to inter-quartile range; thin horizontal lines indicate range of observed values. Only the significant differences are reported.

The intra-specific analysis on play initiation as a function of the playmates' ages revealed interesting differences between the two species. In chimpanzees, there was a significant difference in play initiation frequency among the three age-classes considered (Kruskall-Wallis' χ^2^  = 23.112, N_I_ = 8, N_J_ = 7, N_A_ = 21, d.f. = 2, p = 0.00001). Adults initiated play bouts less frequently than juveniles and infants (Dunn's post-hoc test; N_I_ = 8, N_A_ = 21, Q = 4.34, p = 0.0001; N_J_ = 7, N_A_ = 21, Q = 3.47, p = 0.0002), whereas no difference was found between infant and juvenile subjects (Dunn post-hoc test: N_I_ = 8, N_J_ = 7, Q = 0.298, n.s.) (Figure 3a). Also in bonobos, the analysis showed a significant difference in play initiation rates across the three age classes (Kruskall-Wallis' χ^2^  = 15.789, N_I_ = 7, N_J_ = 6, N_A_ = 21, d.f. = 2, p = 0.00001). Juveniles initiated play bouts more frequently than adults (Dunn post-hoc test N_J_ = 6, N_A_ = 21, Q = 4.14, p = 0.0001); whereas, no difference was found between infants and juveniles (Dunn post-hoc test: N_I_ = 7, N_J_ = 6, Q = 1.95, n.s.) and between infants and adults (Dunn post-hoc test: N_I_ = 7, N_A_ = 21, Q = 1.55, n.s.) (Figure 3b).

#### Polyadic play frequency

**Figure 4:**
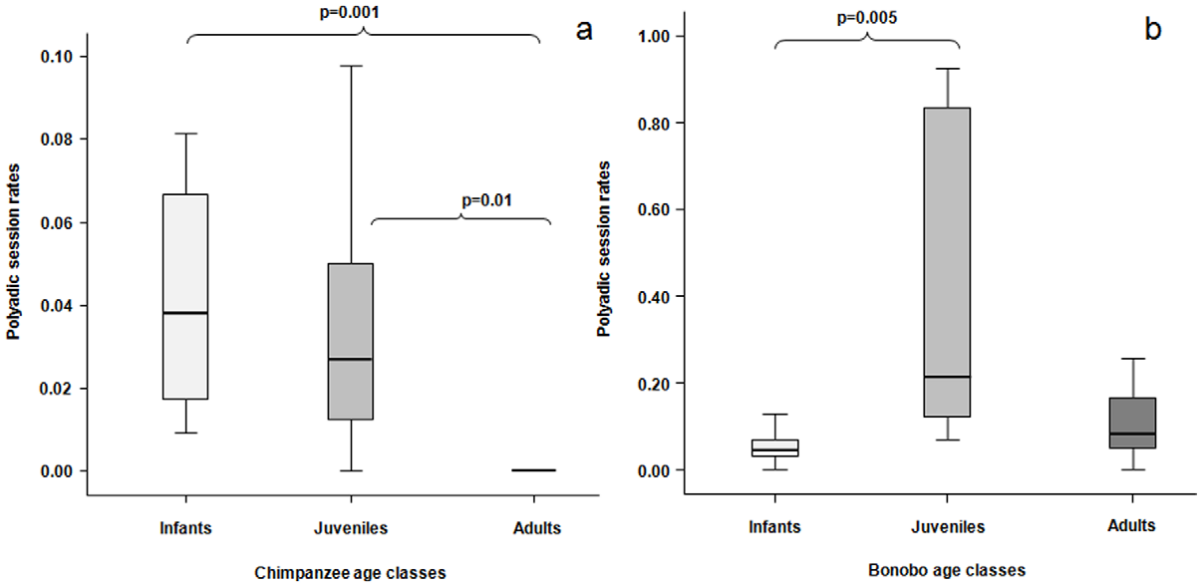
Polyadic play interactions of bonobos and chimpanzees. Rates of the polyadic play sessions performed by the three age classes of chimpanzees (a) and bonobos (b). Solid horizontal lines indicate medians; length of the boxes corresponds to inter-quartile range; thin horizontal lines indicate range of observed values. Only the significant differences are reported.

For each play session, the number of playmates was also recorded, thus permitting the distinction between dyadic (two players involved) and polyadic (more than two players involved) play sessions (as described by Hayaki [24]). The individual rates of polyadic sessions were defined as the number of polyadic sessions divided by the total number of play sessions performed. The polyadic session frequency negatively correlated with the ages of chimpanzees (r_s_ = −0.705, n = 36, p = 0.000001) but not with those of bonobos (r_s_ = 0.121, n = 34, p = 0.496). In chimpanzees, the analysis revealed a significant difference in polyadic play levels across the three age classes (Kruskall-Wallis' χ^2^  = 23.33, N_I_ = 8, N_J_ = 7, N_A_ = 21, d.f. = 2, p = 0.00001). Infant and juvenile chimpanzees did not differ in their polyadic play session rates (Dunn post-hoc test: N_I_ = 8, N_J_ = 7, Q = 0.54, n.s.); adult chimpanzees performed significantly less polyadic sessions than infants (Dunn post-hoc test N_I_ = 8, N_A_ = 21, Q = 3.79, p<0.001) and juveniles (Dunn post-hoc test N_J_ = 7, N_A_ = 21, Q = 2.96, p<0.01) (Figure 4a).

In bonobos there was a significant difference in polyadic play rates across the diverse ages (Kruskall-Wallis' χ^2^  = 8.774, N_I_ = 7, N_J_ = 6, N_A_ = 21, d.f. = 2, p = 0.012). Juvenile bonobos performed polyadic sessions more frequently than infants (Dunn post-hoc test N_I_ = 7, N_J_ = 6, Q = 2.96, p = 0.005). Polyadic session rates of adult bonobos were comparable to those of infants (Dunn post-hoc test N_I_ = 7, N_A_ = 21, Q = 1.61, n.s.) and juveniles (Dunn post-hoc test N_J_ = 6, N_A_ = 21, Q = 2.03, n.s.) (Figure 4b).

#### Play session length

We measured the length (seconds) of each play session per each dyad. To estimate the individual session length, we calculated the median of the medians of the length of the sessions in which that subject was involved.

Both in chimpanzees and bonobos we did not find any correlation between the play length of each session and the subjects' ages (chimpanzees: r_s_ = −0.134, n = 36, n.s.; bonobos: r_s_ = −0.380, n = 34, n.s.). The duration of each play session did not differ between bonobo and chimpanzee infants (U = 8.00, n_chimp_ = 8, n_bon_ = 7, n.s.; mean duration of each session, bonobos = 4.91±0.55 SE; chimpanzees = 3.25±0.19 SE) and between bonobo and chimpanzee adults (U = 182.00, n_chimp_ = 21, n_bon_ = 21, n.s.; mean duration of each session, bonobos = 2.93±0.18 SE; chimpanzees = 3.71±0.39 SE). As for juveniles, bonobos showed a longer duration of each session compared to chimpanzees (U = 3.00, n_chimp_ = 7, n_bon_ = 6, p = 0.007; mean duration of each session, bonobos = 5.40±0.68 SE; chimpanzees = 3.06±0.24 SE).

#### Escalation of play into overt aggression

Play sessions can sometimes escalate into overt aggressions. In the two *Pan* species, there are some vocalizations and facial displays that express fear such as screaming and bared-teeth [25]. We classified as “escalated” those play sessions that ended with screaming and/or bared-teeth by one of the players and/or ended with an aggressive interaction (e.g., chase/flee) between them. We measured the individual frequency of escalation as the number of escalated play sessions on the number of the total play sessions performed by each subject.

**Figure 5:**
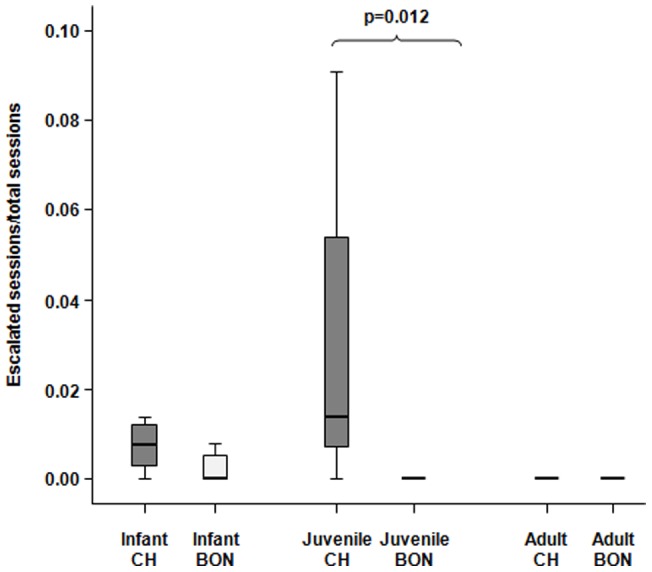
Escalation of social play into aggressive encounters. Rates of the escalated play sessions performed by infants, juveniles, and adults of the two *Pan* species. Solid horizontal lines indicate medians; length of the boxes corresponds to inter-quartile range; thin horizontal lines indicate range of observed values. Only the significant differences are reported.

Bonobo and chimpanzee infants did not differ in the rates of escalated play sessions (Mann-Whitney's U = 14.00, n_chimp_ = 8, n_bon_ = 7, p = 0.103). A similar result was also found when we compared the adults of the two species (U = 210.0, n_chimp_ = 21, n_bon_ = 21, p = 1.0). On the other hand, juvenile chimpanzees, compared to juvenile bonobos, showed higher levels of play escalation into overt aggression (U = 4.50, n_chimp_ = 7, n_bon_ = 6, p = 0.012) (Figure 5).

## Discussion

The divergence of play developmental timing between bonobos and chimpanzees seems to occur during the transitional phase from infancy to juvenility. This divergence emerges particularly in social play, which shows greater variation in the two *Pan* species.

Many authors agree that solitary play, in its object and acrobatic version, has similar adaptive functions in social animals [20], [26]–[28]. For this reason, it is not surprising that the two *Pan* species share similar developmental pathways in the motivation to engage in this activity. Many scholars affirm that object and acrobatic play, independently of different cultures [29], [30], helps individuals in developing cognitive [31] and physical skills [32], which are relevant to subsistence activities such as prey catching, agonistic behavior, and tool use [26], [33]. For example, human and non-human acrobatic play, with its balance-disturbing actions (e.g., somersaults, pirouettes, body-rotation), provides an important vestibular stimulation, which favors motor development [34]. The higher frequency of acrobatic play recorded for juvenile bonobos compared to chimpanzees can be related to the delay in the body size development of skeleton features [10], [35] and locomotion habits of *Pan paniscus* [36]. In fact, with their varied locomotion involving arboreal quadrupedal and bipedal activities, bonobos are considered the most suspensory of the African apes [37].

The most striking divergence in the developmental pathways of bonobos and chimpanzees are evident in a particular type of social play: play fighting. Chimpanzees engaged in less play fighting sessions as their age increased, in contrast with bonobos, who maintained constant levels of play throughout infant, juvenile, and adult periods. The hotspot for play fighting timing divergence is juvenility; in fact, infant bonobos and chimpanzees showed similar levels of this practice, which began to follow a divergence trend at the onset of the juvenile phase. Play fighting is one of the most complex interactions used by human and non-human animals to gather information on the potential of conspecifics as competitors or social partners [19]. This competitive/cooperative interaction serves to test a partner's willingness to invest in a relationship and, simultaneously, to demonstrate one's own willingness to accept vulnerability [6].

Compared to chimpanzees, social play sessions in juvenile bonobos escalated less frequently into overt aggression, lasted longer, and frequently involved more than two partners concurrently (polyadic play). All these findings suggest that social play can undergo a functional shift from infancy to juvenility. In juvenile bonobos, play fighting seems to maintain a cooperative mood [21], [23], whereas in juvenile chimpanzees [38] and in human adolescents [20], [39] it acquires more competitive elements. A further, but not alternative, explanation for the divergence trend in social play of the two *Pan* species could be the low degree of bonobo social inhibitory control, which is essential to make this practice efficient [19]. Bonobos, compared to chimpanzees, show a developmental delay in social inhibition that can be responsible for the retention of juvenile traits into adulthood [15]. Our data on social play go further by indicating that in bonobos such delay can be responsible for the retention of infant traits into the juvenile period. Play ontogenetic pathways of immature bonobos seem to show similarities with play ontogenetic pathways of children. In fact, even though ethological data are scarce, a human cross-cultural analysis of social play revealed some stylistic variations but a common distribution in frequency according to the different age phases [40].

Both infants and adults of the two *Pan* species showed a similar duration of a single play session, which, on the contrary, differed between the juveniles of the two species, with chimpanzees performing shorter sessions than bonobos. This finding, together with the low preference for chimpanzees to engage in polyadic play, indicates that juveniles of this species are less able than bonobos to manage a playful session in relation to time and number of playmates. This is probably due to the higher competitive nature of chimpanzee playful interactions [39] and to their lower social tolerance degree, which become evident in the juvenile phase [41].

In conclusion, our findings show that the divergence of play ontogenetic pathways between the two *Pan* species and the relative emergence of play neotenic traits in bonobos can be detected before individuals reach sexual maturity. The high play motivation showed by adult bonobos [16] compare to chimpanzees [6] is probably the result of a long developmental process which is rooted in the delicate transitional phase, which leads subjects from infancy to juvenility.

## Methods

### Ethics statement

This study was approved by University of Pisa (Animal Care and Use board). Since the study was purely observational the committee waived the need for a permit. The study was conducted with no manipulation of animals. The parks gave the permission to collect data on the animals.

### The study colonies, data collection, and analysis

The study involved eight infant, seven juvenile and 21 adult chimpanzees (2001–2002, ZooParc de Beauval, France; 2004, Dierenpark Amersfoort, the Netherlands) and seven infant, six juvenile, and 21 adult bonobos (2000–2003, 2009, Apenheul Primate Park, the Netherlands; 2006, FrankfurtZoo, Germany; 2009–2010, WilhelmaZoo, Germany) (Table 2). Since the chimpanzee and bonobo ages did not differ, we could compare the different *Pan* species groups (infants: Mann-Whitney U = 27; n_chimp_ = 8; n_bon_ = 7, n.s.; juveniles: U = 13.5; n_chimp_  = 7; n_bon_  = 6; n.s.; adults: U = 154; n_chimp_ = 21; n_bon_ = 21; n.s.).

**Table 2:** Composition of the chimpanzee and bonobo groups.

Subjects	Sex Class	Age in years	Mother-Offspring Relationship	Residence
**CHIMPANZEE GROUPS**				
Ituri	Female	0.5		Amersfoort
Kumi	Male	2.0		Amersfoort
Karibuna	Male	2.5		Amersfoort
Ghafula	Female	3.5		Amersfoort
Chura	Female	6.0		Amersfoort
Bibi	Female	7.0		Amersfoort
Willy	Female	13.0		Amersfoort
Sanne	Female	15.0		Amersfoort
Cees	Male	25.0		Amersfoort
Belle	Female	27.0		Amersfoort
Silvia	Female	30.0	Sanne's mother	Amersfoort
Jet	Female	34.0		Amersfoort
Sjimmie	Female	37.0		Amersfoort
Kokkie	Female	38.0	Cees'mother	Amersfoort
Sjors	Female	38.0	Ghafula and Ituri's mother	Amersfoort
Mike	Male	39.0		Amersfoort
Sonja	Female	40.0		Amersfoort
Rachel	Female	1.0		Beauval
Bazou	Male	2.0		Beauval
Makury	Male	2.5		Beauval
Melie	Female	3.5		Beauval
Leo	Male	4.0		Beauval
Isabel	Female	5.5		Beauval
Benji	Male	6.0		Beauval
Christmas	Female	6.5		Beauval
Tsavo	Male	7.0		Beauval
Gamin	Male	13.0		Beauval
Domi	Female	13.0	Rachel's mother	Beauval
Gypso	Female	15.0	Melie's mother	Beauval
Joseph	Male	19.0		Beauval
Bonobo	Female	20.0	Benji and Makuri's mother	Beauval
Julie	Female	20.0	Christmas and Leo's mother	Beauval
Baraka	Female	23.0	Tsavo and Bazou's mother	Beauval
Micheline	Female	24.0		Beauval
Charlotte	Female	29.0	Domi and Isabel's mother	Beauval
La Vieille	Female	43.0		Beauval
**BONOBO GROUPS**				
Jasiri	Female	0.5	Lomela's daughter	Apenheul
Kumbuka	Female	1.5	Molaso's daughter	Apenheul
Tarishi	Male	2.5	Jill's son	Apenheul
Liboso	Female	2.5	Zuani's daughter	Apenheul
Hongo	Male	3.5	Hortense's son	Apenheul
Nayembi	Female	3.5	Liboso's daughter	Apenheul
Lingala	Female	6.0	Jill's daughter	Apenheul
Lomela	Female	9.0	Jasiri's mother	Apenheul
Zuani	Female	10.0	Liboso's mother	Apenheul
Hani	Male	11.0		Apenheul
Rosie	Female	11.0		Apenheul
Zamba	Male	11.0	Hortense's son	Apenheul
Molaso	Female	15.0	Kumbuka's mother	Apenheul
Mwindu	Male	15.0		Apenheul
Jill	Female	15.0	Tarishi and Lingala's mother	Apenheul
Mobikisi	Male	20.0		Apenheul
Hortense	Female	31.0	Zamba and Hongo's mother	Apenheul
Kelele	Male	2.0		FrankfurtZoo
Heri	Male	5.0	Natalie's son	FrankfurtZoo
Haiba	Female	5.0	Ukela's daughter	FrankfurtZoo
Kutu	Female	8.0		FrankfurtZoo
Zomi	Female	8.0		FrankfurtZoo
Kamiti	Female	19.0		FrankfurtZoo
Ukela	Female	21.0	Natalie's daughter	FrankfurtZoo
Ludwig	Male	22.0		FrankfurtZoo
Natalie	Female	40.0	Ukela and Heri's mother	FrankfurtZoo
Margrit	Female	54.0		FrankfurtZoo
Kianga	Female	5.0	Kombote's daughter	WilhelmaZoo
Kasai	Male	5.5	Chipita's son	WilhelmaZoo
Banbo	Female	7.0		WilhelmaZoo
Mixi	Female	8.0	Chipita's daughter	WilhelmaZoo
Chimba	Female	14.0		WilhelmaZoo
Chipita	Female	18.0	Mixi and Kasai's mother	WilhelmaZoo
Kombote	Female	43.0	Kianga's mother	WilhelmaZoo

All the study subjects were reared and breastfed by their natural mothers and all were in good health. All the chimpanzee and bonobo groups were housed in enclosures made of both indoor and outdoor facilities equipped with trunks, lianas, ropes, stones, and platforms so the animals could move freely in all three dimensions. The visibility conditions were excellent for each of the study groups considered. Both chimpanzees and bonobos were fed three times a day with vegetables, fresh fruits, nuts, grains, eggs, pellets, and yogurt that were scattered on the ground or concealed under trunks or stones. No animals performed any sign of distress or stereotypic behaviors.

Before systematic data collection, the eight observers underwent a training period to become skilled in play patterns and animals' identification. For each observer involved in this study, the training was carried out by the first author. Each training session was performed on the colony the observers would follow for data collection. During the training process, after recording data on the same play session the observers compared and contrasted the collected items. This procedure permitted a measure of inter-observer reliability by calculating the Cohen's kappa value, which was calculated at least three times during the entire observational period for each colony. Cohen's kappa values were never less than 0.70. Only Elisabetta Palagi looked at all the groups involved in the study, whereas each other observer looked at only one group. Observations took place over a 6-hour period, 6 days per week (also covering the feeding-times). Via focal sampling (individual mean hours 31±1.40 SE for chimpanzees; 40±0.92 SE for bonobos) we collected data on solitary and social play.

Due to the non-normality of the data and the small sample sizes, nonparametric statistical tests were used for the analyses at the individual level [42]. We made use of exact tests according to the threshold values suggested by Mundry & Fischer [43]. For all the two-tailed analyses, we adjusted the significance level via the Bonferroni correction (α/3 = 0.017). The analyses were performed by using SPSS 12.0.
